# Carbon Anode Materials for Rechargeable Alkali Metal Ion Batteries and *in-situ* Characterization Techniques

**DOI:** 10.3389/fchem.2020.607504

**Published:** 2020-12-17

**Authors:** Ruida Ding, Yalan Huang, Guangxing Li, Qin Liao, Tao Wei, Yu Liu, Yanjie Huang, Hao He

**Affiliations:** ^1^College of Materials Science and Engineering, Changsha University of Science & Technology, Changsha, China; ^2^Department of Physics, City University of Hong Kong, Hong Kong, China; ^3^Shenzhen Research Institute, City University of Hong Kong, Shenzhen, China

**Keywords:** ion batteries, carbon anode materials, *in-situ* techniques, solid electrolyte interface, ions intercalation, structural evolution

## Abstract

Lithium-ion batteries (LIBs), used for energy supply and storage equipment, have been widely applied in consumer electronics, electric vehicles, and energy storage systems. However, the urgent demand for high energy density batteries and the shortage of lithium resources is driving scientists to develop high-performance materials and find alternatives. Low-volume expansion carbon material is the ideal choice of anode material. However, the low specific capacity has gradually become the shortcoming for the development of LIBs and thus developing new carbon material with high specific capacity is urgently needed. In addition, developing alternatives of LIBs, such as sodium ion batteries and potassium-ion batteries, also puts forward demands for new types of carbon materials. As is well-known, the design of high-performance electrodes requires a deep understanding on the working mechanism and the structural evolution of active materials. On this issue, *ex-situ* techniques have been widely applied to investigate the electrode materials under special working conditions, and provide a lot of information. Unfortunately, these observed phenomena are difficult to reflect the reaction under real working conditions and some important short-lived intermediate products cannot be captured, leading to an incomplete understanding of the working mechanism. *In-situ* techniques can observe the changes of active materials in operando during the charge/discharge processes, providing the concrete process of solid electrolyte formation, ions intercalation mechanism, structural evolutions, etc. Herein, this review aims to provide an overview on the characters of carbon materials in alkali ion batteries and the role of *in-situ* techniques in developing carbon materials.

## Introduction

The energy crisis and severe environmental issues have driven scientists to develop efficient energy conversion solutions to utilize renewable energy and reduce emissions (Armand and Tarascon, 2008; Winter et al., 2018). Although traditional lead-acid batteries play an irreplaceable role in society, they are insufficient to meet the requirement of energy density for electronic devices and electric vehicles. Therefore, it is urgent to develop a more efficient and lighter energy storage system. A high energy density Li-based battery (e.g., Li-LiTiS_2_ cell), developed in the 1970s, was not successfully commercialized due to the safety issue of Li metal as the anode electrode (Whittingham, 1976). With the development of cathode materials and electrolyte systems (Armand and Touzain, 1977; Armand et al., 1979; Mizushima et al., 1981; Yazami and Touzain, 1983), anode materials with a stable and reversible ion intercalation/deintercalation property is urgently needed to form a full ion battery. In 1985, Yoshino et al. developed a full lithium ion battery (LIB) with LiCoO_2_ as cathode, carbon material as anode and LiPF_6_/ethylene carbonate (EC)/propylene carbonate (PC) as electrolyte, respectively. This battery is further commercialized by SONY in 1991 (Yoshino et al., 1987; Yoshino, 2012) and “a reachargeable world was created at this time” (The Nobel Prize in Chemistry, 2019).

With the large-scale promotion of lithium-ion batteries, lithium resources have gradually become one of the most concerning issues. Sodium and potassium, showing similar properties to lithium but with much higher reserves, are considered to be alternatives for lithium (Hou et al., 2017; Pramudita et al., 2017). Since sodium-ion batteries (SIBs) and potassium-ion batteries (KIBs) can learn from the technology and materials for LIBs, they are most expected in rechargeable batteries.

Developing anode materials with a high specific capacity plays an important role in enhancing the energy density of alkali ion batteries (LIBs) (Lee et al., 2016). The alloy and conversion type anode materials (e g., Si) showed high specific capacity, however it is still far away from practical applications due to the huge volume expansion (Sun et al., 2016; Yun Zhao et al., 2019). In contrast, carbon materials, showing excellent electrical conductivity and cycle stability, are considered the most promising anode materials for ion batteries. Therefore, it is of importance to further develop stable carbon materials with a high specific capacity to increase battery energy density (Li et al., 2019). The main limitation for SIBs and KIBs is to develop stable anode materials with high specific capacity that can be intercalated with ions reversibly, since the larger radiuses of sodium and potassium cause the hardness for the intercalations. Therefore, the behavior and mechanism of ion intercalation still need further investigation.

In ion batteries, carbon material undergoes three reaction processes in ion batteries: (1) SEI film formation, (2) structural evolution during ion insertion/extraction and (3) performance degradation. In early days, *ex-situ* characterization techniques have been applied and provided a lot of information on the morphology, structure, and composition, and these work play an important role in understanding the working mechanism of active materials in ion batteries. However, it is also found that the *ex-situ* techniques cannot capture the intermediate products and phenomenon during working under high charge/discharge rates. Different from the *ex-situ* techniques, the *in-situ* characterization techniques monitor the signal of active materials in ion batteries under working conditions, providing time-resolved information on solid electrolyte formation, ions insertion/extraction and structural evolutions, etc. (Harks et al., 2015; Yang et al., 2016; Yuan et al., 2017; Zhu et al., 2019). This operando information is of great significance in developing new electrode materials and improving the performance of batteries.

This review aims to highlight the recent developments and discoveries on carbon materials for alkali ion batteries and provide an overview on employing *in-situ* techniques in the understanding the working mechanism. In detail, carbon materials as well as their reaction mechanism in alkali metal ions batteries are elaborately expounded, followed with a presentation about the *in-situ* techniques on the investigation of SEI formation, ion insertion/extraction mechanism, degradation mechanism, etc. Subsequently, a summary on the current progress toward *in-situ* techniques is presented as well as the key issues concerning the challenges and perspectives prospect, which give constructive suggestions on the research focus and direction for developing better carbon anode materials.

## Carbon Materials as Anodes for Ion Batteries

For anode materials in ion batteries, carbon-based materials, silicon-based materials, tin-based materials, and metal oxides have been developed. And after several years of development, carbon materials have become the most successful anode materials. There are usually three types of orbital hybridization for C atoms in carbon materials, e.g., sp, sp2, and sp3. Generally, carbon materials based on sp or sp3 hybrid orbitals, e.g., polyacetylene, hydrocarbons, are difficult materials with which to form a regular structure allowing the insertion/extraction of guest ion, thus they are rarely used as anode materials for ion batteries. The carbon material with sp2 hybridization takes a planar sheet of carbon atoms in a honeycomb structure as a basic unit, and is formed by a stack of these basic units in an ordered or disordered manner (Wu et al., 2003). These three orbital hybrid carbon atoms form a large amount of carbon materials with diverse structures which can be characterized by X-ray diffraction (XRD), Raman, Transmission electron microscopy (TEM), Nitrogen sorption measurements, etc. (Xing et al., 2017b), and these diverse structures ensure their application in energy storage and conversion (Xing et al., 2015, 2016, 2017a). This structure information is generally characterized by Alkali metal ions, such as lithium, sodium, and potassium, and can reversibly intercalate into these materials. This section will focus on these carbon materials.

### Types of Carbon Anode Materials

#### Graphite

**Figure 1:**
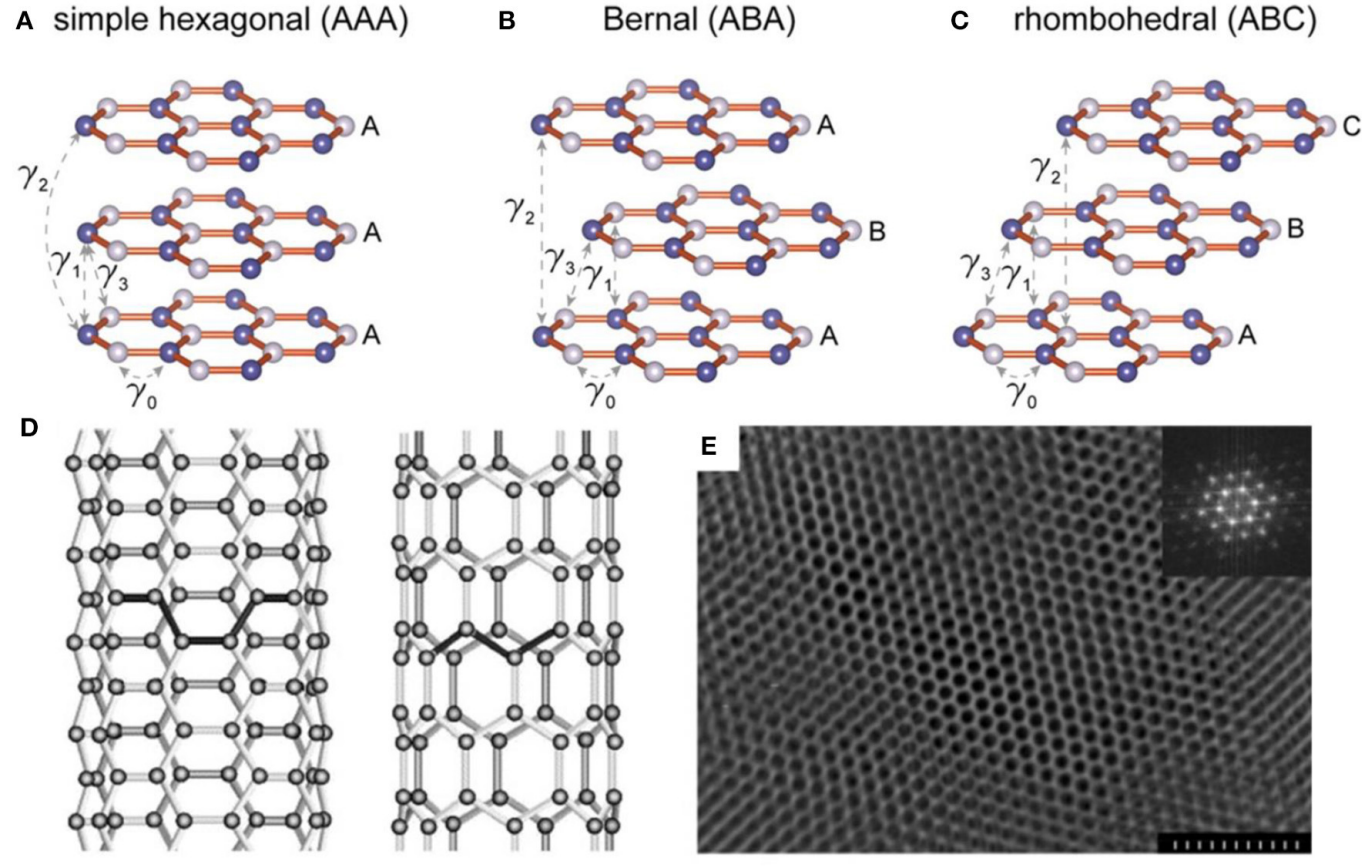
Three types of stackings for layered graphene and the corresponding calculated electronic structures. **(A)** Schematic drawings of simple hexagonal (AAA), **(B)** Bernal (ABA), and **(C)** rhombohedral (ABC) stackings (Bao et al., 2017). **(D)** Illustrations of the atomic structure of (left) an armchair and (right) a zig-zag single-walled nanotube (Thostenson et al., 2001). **(E)** Electron microscopy image of the highly ordered mesoporous carbon structure. In the inset, a Fourier transform (FT) of the image shows a pattern with multiple reflections, which are characteristic of a highly ordered hexagonal array. The scale bar is 300 nm (Liang et al., 2004).

Graphite, one of the most widely used anode materials in commercial LIBs, delivers a capacity of 372 mA h g^−1^ which corresponds to the formation of LiC_6_ (Hou et al., 2017). Due to the high initial coulombic efficiency (CE), low volume expansion, rich natural resources and the ability to form graphite intercalation compounds (GICs), graphite is also widely applied as an anode material in potassium ion batteries (PIBs) and other alkaline metal ion batteries, such as Rb and Cs ion battery (Dresselhaus and Dresselhaus, 2002). As is well-known, the structure of graphite is a unique layered structure with hexagonal stacking (AAA), Bernal stacking (ABA), or rhombohedral stacking (ABC), as shown in Figures 1A–C (Bao et al., 2017). The structure features a strong covalent bond within the graphene layers with a weak bond through van der Waals interactions in the vertical direction, resulting in an interlayer spacing of 3.35 Å which is capable of guest ions insertion/extraction. This structure ensures that alkali metal ions can be inserted into graphite to form metallized graphite. Generally, the layered structure of graphite will not be destroyed during the ion insertion/extraction, which ensures its long-term cycle performance in ion batteries. Conversely, anodes based on alloys or conversion reactions exhibit severe volume changes during cycling, which can easily lead to electrode material failures. Therefore, due to the high stability and long cycle life, it is reasonable to use graphite as the preferred anode based on intercalation chemistry.

For LIBs, graphite is one of the most widely used anode materials. Due to its good electrical conductivity, high crystallinity, and good layered structure, lithium ions can be reversibly inserted or extracted, and a theoretical specific capacity of 370 mAhg^−1^ and an efficiency above 90% are delivered. The intercalation reaction of lithium in graphite occurs at 0–0.25 V (vs. Li/Li+), which makes graphite an anode that can be matched with a variety of positive electrode materials to form a battery with high voltage. Similar to lithium, potassium ions also undergo reversible intercalation/extraction reactions in graphite, resulting in a reversible specific capacity of 273 mAhg^−1^ (Jian et al., 2015).

Different from LIBs and potassium ion batteries (PIBs), graphite is considered to be difficult to apply as an anode for sodium ions batteries (SIBs). The reason leading to this issue is attributed to the fact that the insertion mechanisms of different alkali ions into graphite are different, so the electrochemical behavior of graphite electrodes in LIBs, SIBs, and PIBs varies a lot. Unfortunately, the fundamental origin of the difference is still not clear due to the complexity of the system and the difficulty of quantifying ion intercalation (Winter et al., 2018). To explore the cause of the undesirable Na^+^ storage properties of graphite, the mechanism of Na^+^ insertion into graphite has been investigated by theoretical studies. According to the calculations of density functional theory (DFT), it was found that the local binding of a Na ion to a single layer of graphene was quite unstable by ≈0.5 eV (*E*_i_) compared with those for other alkali metals (AMs). This *E*_i_ was in accordance with the formation energy (*E*_f_) reported for AM-GICs (Nobuhara et al., 2013; Okamoto, 2014; Wang et al., 2014; Liu et al., 2016; Yoon et al., 2017) (SM 50), which revealed that the repulsive local interactions between graphite layers and Na^+^ ions dominantly destabilized the Na-GICs, consequently leading to poor sodium storage capacities in graphite anodes. The poor performance of graphite anodes on sodium storage has been preliminarily revealed through the theoretical calculation, however, there is still a lack of experimental evidence. Investigation of sodium ions intercalation behavior in graphite by multiple *in-situ* techniques is of great significance, and the results will provide guidance for developing new carbon anode materials for SIBs.

#### Carbon Nanotube (CNT)

As one of the carbon allotropes, carbon nanotubes (CNTs) have been approved to be an additive or substitute in LIBs anode owing to the chemical stability, large surface area, robust mechanical properties, and high electrical conductivity (Thess et al., 1996; Gu et al., 2015; Liew et al., 2015). The structure of CNTs presents as one-dimensional cylindrical tubule of graphite sheets, including multi-walled (MWCNTs), double-walled (DWCNTs), and single-walled (SWCNTs) tubes (Figure 1D). This tubular structure contributes to the mitigation of structural integrity degradation resulting from the significant volume change during charge/discharge process. In addition, CNTs possess superb conductivity (~10^6^ S m^−1^), high rigidity (Young's modulus of the order of 1 TPa), low density and high tensile strength (60 GPa) (Thess et al., 1996; Treacy and Ebbesen, 1996; Lier et al., 2000; Yu et al., 2000). In LIBs, SWCNTs display a reversible capacity ranging from 300 to 600 mAhg^−1^, significantly higher than that of graphite (372 mAhg^−1^), making them a competitive lithium storage material (Meunier et al., 2002; Yang et al., 2008).

Generally, the reversible capacity can reach up to 1,000 mAhg^−1^ via mechanical and chemical treatments. To achieve this purpose, one of the typical ways is to synthesize hybrid composites with CNT as a key component. For example, Su and his co-workers demonstrated a lightweight CNT paper as a freestanding framework to accommodate Li metal in a mass fraction of 80.7 wt% (Sun et al., 2018). The highly conductive CNT network effectively inhibited the formation of Li dendrites so that the anode afforded a favorable coulombic efficiency of over 97.5%. Additionally, owing to the robust and expandable nature of the CNT paper beyond other 3D scaffolds, the Li/CNT anodes exhibited areal and gravimetric capacities of 10 mA h cm^−2^ and 2,830 mA h g^−1^. The Li utilization after 1,000 cycles at a current density of 10 mA cm^−2^ remained 90.9%. Inserting the substitutional heteroatoms in the graphitic layers is another way to optimize the properties of CNTs in ion batteries. Due to their similar sizes, N and B are the most preferred heteroelements to replace C atoms. A novel aligned N-doped core-sheath carbon nanotube (N-CNT) film has been synthesized by Pan et al., and a high capacity of 390 mAh g^−1^ and retention of 97% after 200 cycles at a rate of 4C were delivered in LIBs (Pan et al., 2016). It is found that nitrogen-doped graphene shells could facilitate the insertion of lithium ions.

Nonetheless, the capacity of CNTs are partially irreversible in LIBs since a fraction of the inserted lithium during the first charge process is consumed in formation of SEI. Another problem for CNT-based anodes is the lack of voltage plateau during discharging process. The broad changes in voltage make its utilization difficult in most electronics which require a stable voltage source. In fact, all the disadvantages of CNT are related to morphology. Therefore, *in situ* characterization methods, such as *in-situ* TEM, Raman, FTIR, etc., are needed to conduct multi-scale observation on CNT anode materials to figure out the relationship between their structure and properties. This information is of important guiding significance for the researchers to develop new synthesis methods and novel carbon nanotubes.

#### Amorphous Carbon Materials

Amorphous carbon materials, including soft carbon materials and hard carbon materials, are short of long-range ordered structure in plane and ordered stacked structures of graphite. Generally, the amorphous carbon materials are comprised of voids, distorted graphene nanosheets, and randomly distributed graphitized micro-domains, so they prefer to remain in the amorphous structure and restrain the development of graphitic structure.

Soft carbon, including petroleum coke, needle coke, carbon microspheres, etc., can be transformed into graphite after heat treatment at a temperature above 2,000°C. Due to the small crystal grain size and large interplanar spacing, soft carbon materials show a faster lithium ion diffusion coefficiency and a more stable charge/discharge platform during lithiation/delithiation.

In contrast, hard carbon maintains a disordered structure permanently regardless of the temperature (Bommier and Ji, 2015). This is mainly due to the formation of three-dimensional crosslinking via sp3 hybridization at the early stage of carbonization, which hinders the parallel growth of the graphite surface. Generally, hard carbon is prepared by pyrolysis of polymers such as resin and organic polymer. The pore structure and good electrolyte compatibility of hard carbon ensure it to be a potential storage material for AM ions. Unfortunately, hard carbon materials show a large irreversible capacity decay during the first charge-discharge.

The interlayer spacing for d002 is 3.4–3.6 and 3.7 Å for soft carbons and hard carbons, respectively, which is bigger than that of graphite (3.35 Å) (Saurel et al., 2018). Therefore, amorphous carbon materials, especially hard carbon materials, are excellent anode materials for AM-ion batteries due to their being kinetically favorable for the transportation of ion and electron. Many micro/nanostructured hard carbon materials have been exploited as anode materials for LIBs, such as hollow nanostructured carbon materials (Cao et al., 2012; Tang et al., 2012), porous carbon materials (Yan et al., 2014; Hou et al., 2015), carbon fibers (Bai et al., 2015; Zhu et al., 2015), carbon nanosheets (Ding et al., 2013; Wang et al., 2013; Yang et al., 2015), etc. In addition to being directly used as electrode materials, these carbon materials can be prepared as compounds by heteroatomic doping, loading with metal oxides, etc. to enhance their electrochemical performances. For instance, Zheng et al. reported an anode material obtained by coating the lithium metal on a monolayer of interconnected amorphous hollow carbon nanospheres, which showed excellent performance in LIBs (Zheng et al., 2014). Due to the protection of carbon materials, lithium dendrites did not form up and thus the composite displayed a high Coulombic efficiency of ~99% after more than 150 cycles. Yang et al. synthesized N-doped hollow carbon nanospheres with an optimized shell thickness of 20 nm and N dopant concentrations of 16.6 at %. This anode material delivered a specific capacity of 2,053 mA h g^−1^ at 100 mA g^−1^ and a superior cycling stability of 879 mA h g^−1^ at 5 A g^−1^ after 1,000 cycles (Yang et al., 2017).

Although hard carbon materials are widely used as electrode materials in ion batteries, the *in situ* characterization on them is still rarely reported since the structure changes of amorphous structures are difficult to capture by multiple detection techniques. This is one of the major challenges in developing hard carbon as anode material for ion batteries.

#### Mesoporous Carbon Materials

Compared with block carbon materials, porous carbon materials deliver a significant enhancement in power density and energy density and they have attracted wide attention regarding their employment as electrode materials of energy storage devices. Porous carbon materials display many advantages, such as high surface area, numerous active sites for lithium ions adsorption and storage and pore sizes ranging from nanometers to microns. Figure 1E shows the electron microscopy image of the highly ordered mesoporous carbon structure. These typical morphological features enable porous carbon materials, continuous electron conduction pathways, high-speed ion migration channels, large electrolyte/electrode interface, strain relaxation during the charge/discharge process and high electrical conductivity and thus an enhanced energy storage performance (Hu et al., 2007; Woo et al., 2007; Cheng et al., 2008; Mao et al., 2012). In fact, carbon materials with different pore sizes demonstrate different performances. Microporous carbons (pore size < 2 nm) show greater capacity than conventional graphite. However, they are subject to high irreversibility during lithium insertion/extraction due to their increased solid electrolyte interface (SEI) area and/or the interactions of lithium ions with carbon surface functional groups (Kim et al., 2013). In contrast, mesoporous carbon materials deliver a better capacity reversibility in LIBs.

These high specific surface area carbon materials are usually synthesized by the template method, typically involving the establishment of a sacrificial porous silicon template, followed by impregnation and subsequent carbonization of proper carbon precursors to form a carbon/template composite, and then removing the silicon template to obtain a highly porous carbon. Kim et al. successfully synthesized hollow core-mesoporous shell carbon (HCMSC) with hierarchical nanoarchitecture, and this anode material exhibited a ultra-high Li storage capacity of 1,000 mAh g^−1^ and excellent cycling performance in LIBs (Kim et al., 2011). These improved performances are mainly attributed to the unique structural characteristics, such as large surface area (2,418 m^2^ g^−1^) and mesopore volume of the HCMSC, which facilitated fast mass transport. An ultra-thick mesoporous carbon with a thickness up to 850 μm and an areal mass of 55 mg cm^−2^ was firstly demonstrated by Shen and his co-workers. This mesoporous carbon showed a high specific capacity of 270 mA h g^−1^ and a high areal capacity of 13.6 mA h cm^−2^ in sodium ion half cells, which were significantly higher than those of the state-of-the-art sodium batteries (Shen et al., 2016).

In recent years, the Li storage capacity of porous carbon materials has been further promoted by means of composite with metals or metal oxides or doped with heterogeneous ions (Jahel et al., 2015; Liu H. et al., 2015; Wang et al., 2015; Zhang et al., 2015). For instance, Jahel et al. designed a SnO_2_/C composite with ultra small SnO_2_ particles (~2.0 nm) homogeneously confined in the micro/mesoporous pores of porous carbon to accommodate the volume changes upon lithiation/delithiation. The as-prepared composite delivered a high initial reversible charge capacity of 916 mAh g^−1^ and an reversible capacity retention of 79% after a long cycling life (≈2,000 cycles) (Jahel et al., 2014).

It is well-known that the high specific surface area of carbon material leads to the initial coulombic efficiency of <40% and the changes of the structure and volume during the cycling have great influence on the performance. These issues are the greatest resistance to further applying porous carbon materials in ion batteries. So far, there are few systematic studies on the influence of the porous nanostructure on these phenomena, especially the dynamic evolution of the structure during long-term cycle. Therefore, the introduction of *in-situ* characterization is of great significance to the development of porous carbon materials.

#### Biomass Derived Carbon Materials

Producing carbon materials from biomass materials is theoretically feasible since most biomass materials are mainly composed of C, H and O elements. Additionally, biomass materials are renewable, abundant, and environmentally friendly resources, the application of these sustainable materials is quite attractive (Wang et al., 2017). Experimental results show that, in general, biomaterials typically release some small molecules (e.g., CH_4_, CO, H_2_) during the pyrolysis process, and then convert to amorphous carbon through the crosslinking and partial aromatic ordering process. In the past few years, biomass materials have developed rapidly. A large amount of biomass materials, such as carbohydrates, lignin, chitin, cellulose, proteins, etc., have been prepared as carbon materials and used as electrode materials for energy storage devices. Carbon nanoparticles were prepared by Gaddam et al. through a flame deposition method with coconut oil as carbon sources, and delivered capacities of 277 and 741 mA h g^−1^ in sodium and lithium ion batteries, respectively (Gaddam et al., 2016). Porous hard carbon materials were synthesized by Hong et al. through a simple pyrolysis of H_3_PO_4_-treated pomelo peels at 700°C in N_2_ (Hong et al., 2014). The as-prepared carbon material displayed a 3D connected porous structure and a large specific surface area of 1,272 m^2^ g^−1^. This porous structure ensures a good cycling stability and rate capability. In a sodium ion battery, it delivered a capacity of 181 mA h g^−1^ at 200 mA g^−1^ and the capacity retained 71 mA h g^−1^ at 5 A g^−1^ after 220 cycles.

The biomass-derived carbon shows broad application prospects, and one of the challenges in this field is to develop controllable methods to adjust the functionality of carbon. At present, establishing the relationship between the structure and properties of biomass-derived carbon to guide the improvement of material characteristics (including morphology, porosity, and surface chemistry properties), and achieving the controllable preparation of materials are of great significance for the development of biomass carbon materials.

In addition to the above carbon materials, some new types of carbon materials with special structure are synthesized and applied as anode for ion batteries. Xing et al. synthesized polynanocrystalline graphite via chemical vapor deposition on a nanoporous graphenic carbon as an epitaxial template. This new carbon shows a structure of essentially hollow to a certain extent with randomly arranged nanosized graphite building blocks, which is different to most low-dimensional nanocrystalline carbon materials. This novel structure with disorder at nanometric scales but strict order at atomic scales enables substantially superior long-term cycling life and capacity retention for K-ion storage than that of graphite (Xing et al., 2017c).

### Reaction Process and Mechanism of Carbon Anode Materials in Ions Batteries

#### Ions Storage Mechanism

For lithium-ion batteries, there are usually three storage mechanisms for lithium ions: (i) Li-alloy reaction mechanism, (ii) conversion reaction mechanism that involves the Li oxides (Li_2_O) formation/decomposition as well as the reduction/oxidation of anode material, and (iii) insertion/extraction reaction mechanism that involves the insertion/extraction of Li into/from the lattice of the carbon material (Ji et al., 2011). The former two are usually employed by metal oxides or metals, and the last one is employed by carbonaceous materials such as graphite and CNTs. In order to figure out the mechanism of lithium intercalation into graphite, numerous studies have been conducted (Zanini et al., 1978; Dahn, 1991; Ohzuku et al., 1993). After lithium is intercalated into graphite, lithium atoms will occupy the sites between two adjacent graphene planes, and are associated with a hexagonal C ring in a plane, thus avoiding the nearest neighbor occupation (maximum composition LiC_6_) (Winter et al., 1998). Theoretical studies revealed that the structure of graphite after lithiation fully changes to AA stacking, and the interplanar distance is enlarged from 3.35 Å for x = 0 in Li_x_C_6_ to 3.70 Å for x = 1 (Boehm and Banerjee, 1992; Song et al., 1996). Shi et al. show that both hexagonal and rhombohedral graphite phases are capable of reversible lithium intercalation with little difference in the insertion capability (Shi, 1996; Shi et al., 1997). In fact, the insertion of lithium into graphite conducts through a stage mechanism in which different phases occur sequentially as stage 4, stage 3, stage 2 and stage 1 corresponding to LiC_36_, LiC_24_, LiC_12_, LiC_6_, respectively. The stage mechanism may be attributed to the distinct repulsion of adjacent Li layers which is in competition with lateral interactions, local reactions, and configurational entropy contributions. In charge/discharge curves, different potential plateaus are characteristic of the staging phenomenon, which is also confirmed by the advanced characterization technology such as XRD and Raman spectroscopy.

The AM ion storage mechanism of hard carbon is quite different from the aforementioned carbon materials. The curves of the lithiation/delithiation process is composed of a slopy line in the high potential region and a plateau in the low potential region. To date, there are different opinions on the AM ion storage mechanism in hard carbon anodes. One thinks it is the insertion-absorbtion mechanism, in which the capacity in the slope area mainly results from the insertion/extraction of Li^+^/Na^+^ into/from carbon layers while the capacity in the plateau area is attributed to the adsorption/deposition of Li^+^/Na^+^ in the micropores. For the first time, Stevens and Dahn compared the behaviors of lithium and sodium insertion in hard carbon materials and demonstrated that a similar AM insertion mechanism were followed in both cases (Stevens and Dahn, 2000a). They proposed a “house of cards” model for the carbon structure in which random stacking of multiple layers (2 to 3 layers) creates nanoscale porosity. It is considered that the slope potential profile was attributed to the insertion of lithium or sodium into parallel or nearly parallel layers. Both the interlayer metal content and the random arrangement between parallel slices would affect the lithiation potential. The plateaus at low potential were believed to be attributed to a process analogous to adsorption of metal ions in the nanopores. This process resulted in a potential that was close to the chemical potential of the metal itself, and thus produced an electric potential close to 0 V. Afterwards, *in situ* X-ray scattering was carried out to study the storage of sodium/lithium in hard carbon, proving that both lithium and sodium can be inserted into the interlayer of hard carbon and absorbed in the nanopores. Unfortunately, there are still some experimental phenomena that cannot be fully explained.

In contrast, an absorption-insertion mechanism was proposed, in which the capacity in the slope region was considered to be the absorption of Li^+^/Na^+^ on the surface sites of carbon materials (e.g., functional groups, defective sites and active sites) while the capacity in the plateau region derived from the insertion/extraction of Li^+^/Na^+^. In 2012, Cao et al. investigated the sodium and lithium storage mechanisms in hollow carbon nanowires (HCNWs) and demonstrated that the electrochemical behaviors of HCNWs in SIBs and LIBs are distinctly different (Cao et al., 2012). The sodium storage behavior in HCNWs at the low-potential zone is similar with lithium storage behavior in graphite, corresponding to the intercalation/deintercalation of metal ions into/from graphite interlayers. However, in high-potential area, the electrochemical reaction was believed to be due to the charge transfer on the graphite surface. The theoretical calculation, based on the equilibrium of attractive van der Waals interactions among carbon layers and the repulsion interactions between Na^+^/ Li^+^ and carbon material, was also conducted to investigate the Na^+^/Li^+^ insertion/extraction mechanism for HCNW anodes. The calculation showed that the interplanar equilibrium distances for NaC_6_ and LiC_6_ were 0.45 and 0.37 nm while the energy costs for Li^+^ and Na^+^ insertion were 0.03 and 0.12 eV, respectively. Due to the high energy cost, sodium intercalation into graphite is difficult, however, when the interlayer distance of graphite increased up to 0.37 nm, it could be achieved by overcoming the energy barrier, and this is consistent with the experimental results.

#### Solid Electrolyte Interphase

Solid electrolyte interphase (SEI), a protective layer on the surface of carbon anode, is formed by electrolyte decomposition during the first charge/discharge process. For LIBs, many performances, e.g., the irreversible charge loss, rate capability, cycling performance, graphite anode spalling and the battery safety, are related to the quality of SEI. Generally, the electrolyte of LIBs is thermodynamically unstable at a relatively low or high potential. Thus, there is a reduction process of solvents and salts on the surface of carbon materials, resulting in a deposition of organic/inorganic decomposition to form an SEI membrane. The potential for SEI formation is not a fixed value, generally ranging from 0.5 to 2 V (Ein-Eli et al., 1994; Aurbach et al., 1997; Stevens and Dahn, 2000a; Edström et al., 2006; Bryngelsson et al., 2007; Kang et al., 2008) SEI is mainly formed during the first few cycles and its quality depends on many indicators, such as the additives in the electrolyte, cycling rate, temperature and so on (Ein-Eli et al., 1994; Liebenow et al., 1995). It is desirable that the LIBs are sold to users after a good SEI film has been formed since a well-grown SEI interface would ensure that the dynamic stability of the battery can be improved, and the dynamic stability of electrolyte promoted, thereby ensuring good circulation ability (Aurbach, 2000). The SEI interface also prevents co-insertion of solvents into graphite interlayers, and thereby preventing the exfoliation of graphite anode.

In fact, SEI is a complex layer, and its composition is still not completely determined. At present, it is generally considered to be composed of inorganic components (degradation products of salts in electrolyte) which displays as a dense layer near carbin and organic components (partially or completely reduced products of solvents) which displays as a porous organic or polymer layer (Edström et al., 2006). Additionally, it is difficult to accurately measure the thickness of the SEI since some components are partially soluble in the electrolyte. The thickness of SEI is generally considered to range from a few Angstroms to tens of Angstroms or hundreds of Angstroms. The composition and thickness of the SEI is also considered to be constantly changing during cycling. For example, it may be partially dissolved in the electrolyte [e.g. dimethyl carbonate (DMC)], and the thickness would be larger at low potentials and smaller at high potentials (Winter, 2009).

The parameters of the SEI, such as composition, thickness, shape, and compactness, show significant impact on battery performance. For instance, the irreversible charge loss in the first cycle occurs owing to solvent reduction and the SEI formation (Zaghib et al., 2000). The harmful process (e.g., self-discharge) during the cycle depends mainly on the ability of SEI to passivate the active material, and affects the capacity decay behavior (Yazami and Reynier, 2002). Moreover, the dissolution and transformation of SEI membrane during the cycle also affect the battery performance and stability (Novák et al., 2000). It is also noted that SEI is temperature-sensitive, and thus the performance at high/low temperatures is limited (Churikov, 2001). Most importantly, SEI also has a huge influence on the safety of batteries. Therefore, it is of great significance to understand the properties and behaviors of SEI for the development of better batteries (Park et al., 2009). Unfortunately, the information about SEI is still limited due to the lack of effective characterization techniques.

#### Degradation Mechanism

As mentioned above, a layer of SEI film composed of inorganic and organic substances is formed on the surface of the carbon anode during the first few cycles. And this SEI interface is beneficial to the stability of both carbon anode and organic electrolyte. When lithium ions are intercalated into graphite, the anode potential is very low, which is close to the standard electrode potentials. At such a low potential, the solvent of the electrolyte (e.g., ethylene carbonate) is highly active and could react with lithium to rapidly produce precipitate on the surface of graphite anode (Smith et al., 2012). As the cycle number increases, the thickness of the formed SEI interface increases due to the continued decomposition reaction (Lee and Pyun, 2002; Bodenes et al., 2012). More importantly, the SEI interface retards the kinetics of lithium ions intercalation into carbon anode and is unstable due to the reduplicative expansion and contraction of graphite during lithium insertion and extraction (Li et al., 2001; Aurbach et al., 2006). This reaction leads to the growth of the SEI interface and eventually causes the graphite particles to detach from the current collector. During cycling of LIBs, the formation of this surface membrane is the main cause of lithium ion loss (Broussely et al., 2001). The SEI interlayer also leads to an increase in the charge transfer resistance and blockage of carbon anode, resulting an increase in irreversible capacity (Abraham et al., 2007; Yamada et al., 2008; Moss et al., 2010).

On the other hand, large mechanical strain in graphite would be generated during cycling at high C-rate and high state of charge (SOC), leading to cracks and splits in graphite particles, and thus causing a decrease in orientation (Yuqin et al., 1997). It is reported that the orientation of graphite particles affects the reversible capacity of anode, namely, less-oriented graphite particles display a lower reversible capacity (Shim and Striebel, 2004). This is due to the low orientation of particles, which brings difficulties in kinetics of lithium insertion and leads to the formation of new boundaries between particles through the irreversible interaction of lithium ions and electrolyte (Rhodes et al., 2011; Lai et al., 2012). Furthermore, the content of hexagons changes during cycling while the layered structure of graphite maintains unchanged, and this change substantially leads to the performance degradation (Andersson et al., 1999; Ridgway et al., 2012). Therefore, in order to ensure the stable performance, the content of rhombohedron/hexahedron is an important parameter for carbon anode.

There are several degradation mechanisms for carbon anode in LIBs which seriously affect the long-term cycle life. This degradation can be mitigated by adding various stabilizers, robust electrolytes, and temperature treatments. Thus, it is still necessary to further explore the relationship between the decay mechanism of carbon anodes and external factors, such as electrolytes, voltages, etc., so as to develop an improved stable anode which provides high energy density and good circulability under various operating conditions.

## *In-situ* Techniques for Carbon Materials

Structural characterization of electrode materials is extremely important for understanding the reaction mechanism of the battery during operation. In this regard, the “*ex-situ*” characterization can hardly capture the transient state of structures and the complex electrochemical process involving a series of reactions, thus cannot fully understand the relationship between structural evolution and performance changes of ions batteries. Besides, the *ex-situ* tests normally require the battery to be disassembled and the stripped electrode materials which may react with the trace H_2_O, O_2_, etc. in the glove box, causing irreversible damage, so the test results may not respond to the real state. Moreover, poor consistency is a concern when preparing electrodes at various states, so it is difficult to guarantee that the tested batteries are exactly the same. In addition, numerous batteries should be disassembled for a continuous test, which is really time consuming.

On the other hand, the “*in-situ*” means that the whole characterization is operandoly performed under real working condition, and thus real-resolved signals for electrode materials. The advantages of *in-situ* technique are as follows: (1) the operando evolution of dynamic structure changes under real working condition; (2) focus on the same location to ensure a good comparability among obtained results; (3) collect signals quickly to capture intermediate product information; (4) simple operation.

In this part, the *in-situ* techniques used to investigate carbon materials are summarized, including scanning probe microscopy (SPM), Raman spectra, Fourier transform infrared spectroscopy (FTIR), X-ray/neutron diffraction (XRD/ND), small-angle X-ray/neutron scattering, transmission electron microscope (TEM), and nuclear magnetic resonance spectroscopy (NMR).

### *In-situ* SPM

SPM is an analytical technique for characterizing the real-space morphology of electrodes on the nanometer scale with minimal destruction. One of the most important advantages of SPM is that it can be conducted in a liquid environment to obtain operando information about the SEI film. The edge plane of graphite, which is fault-like, is the main research objective of SPM.

**Figure 2:**
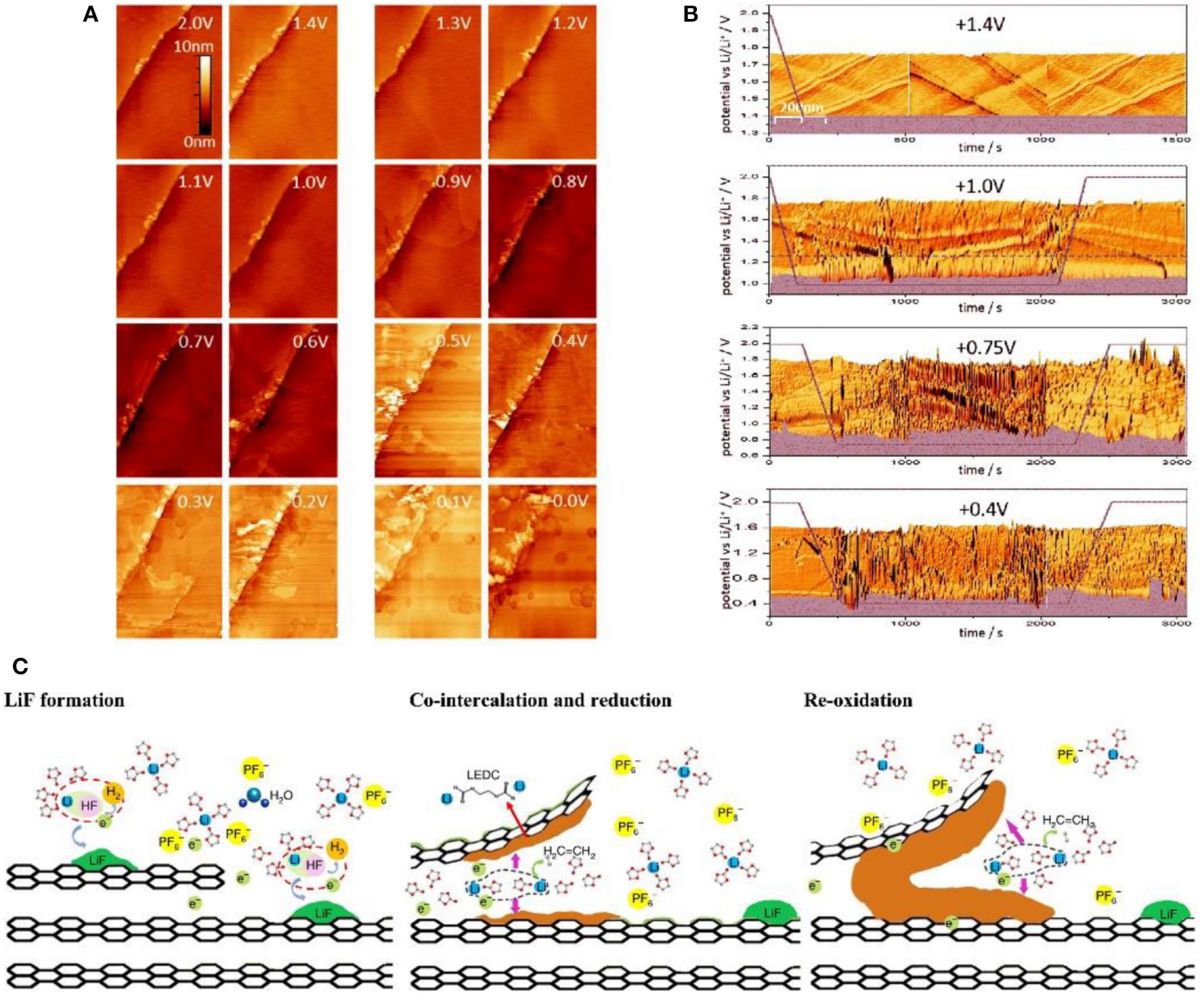
**(A)** STM images of HOPG during delithiation process (Seidl et al., 2016). **(B)** *In-situ* real time STM images of HOPG (Seidl et al., 2016). **(C)** Schematic illustration of the interfacial formation chemistry during the very first lithiation (Liu T. et al., 2019).

STM can visually observe the morphology changes of the graphite edge plane. Combining with atomic force microscope (AFM) which determines the change of the distance between graphite layers, STM can precisely speculate the growth mechanism of SEI film. It is found that SEI film growth mainly includes four stages: (1) HF reduction; (2) intercalation of solvated Li^+^ into graphite; (3) electrolyte reduction on the surface of graphite; (4) enrichment of inorganic phases. The SEI film is initially triggered by electrochemical reaction at the defects and edge plane of graphite at 1.5 V due to the reduction of trace amount of HF to produce LiF particles. The inorganic particles are deposited in a ring-shaped and non-uniform form (Domi et al., 2011). Seidl et al. (2016) employed STM to monitor the SEI formation and found that structural damage began to appear on the graphite edge plane of the highly oriented pyrolytic graphite (HOPG) surface at above 1.0 V (Figure 2A). When the potential decreased to 0.9 V, the irreversible structural change became more pronounced. This phenomenon was further confirmed by AFM at voltage of 0.88 V, wherein the solvated was Li^+^ inserted into the graphite layer and increased the layer space by 1.3 nm or higher (Figure 2C) (Liu T. et al., 2019). At 0.7 V, the graphite edge structure began to break down on a large scale, and the interface structure was more complicated (Figure 2B) (Seidl et al., 2016) since the reduction of solvated Li^+^ produced gel precipitation at 0.74 V (Figure 2C). After this, the electrochemical process is accompanied by the formation of the inorganic layer to form a stable SEI film (Liu T. et al., 2019). The current findings provide a good understanding in the changes of the graphite edge plane during SEI formation, which offers guidance on the formation of a more uniform and stable SEI film.

The common electrolytes used in LIBs cannot guarantee the complete and uniform growth of SEI film, and the thickness of the formed SEI film varies at different locations. Specifically, the thickness of SEI film, measured by AFM, is about 10.4 nm after the first cycle (Xin et al., 2014), and increases by 3.4 nm for the second cycle, demonstrating the non-uniform growth of SEI film (Jeong et al., 2001). Commonly, solvated Li^+^ ions intercalate into the graphite, and pass through the thinner SEI film, thus causing damage to the graphite structure (Jeong et al., 2001). Sometimes, solvated Li^+^ would continuously intercalate into graphene layers, resulting in serious structural deterioration at the edge plane of graphite (Xin et al., 2014; Liu X. -R. et al., 2015). To promote uniform growth of SEI, proper electrolyte additives, e.g., vinyl ethylene carbonate, are added into electrolyte (Domi et al., 2012). It is obvious that *in-situ* observation of SEI growth is of great significance for optimizing SEI, and SPM has unparalleled advantages in this respect.

### *In-situ* Raman Spectra and FTIR

**Figure 3:**
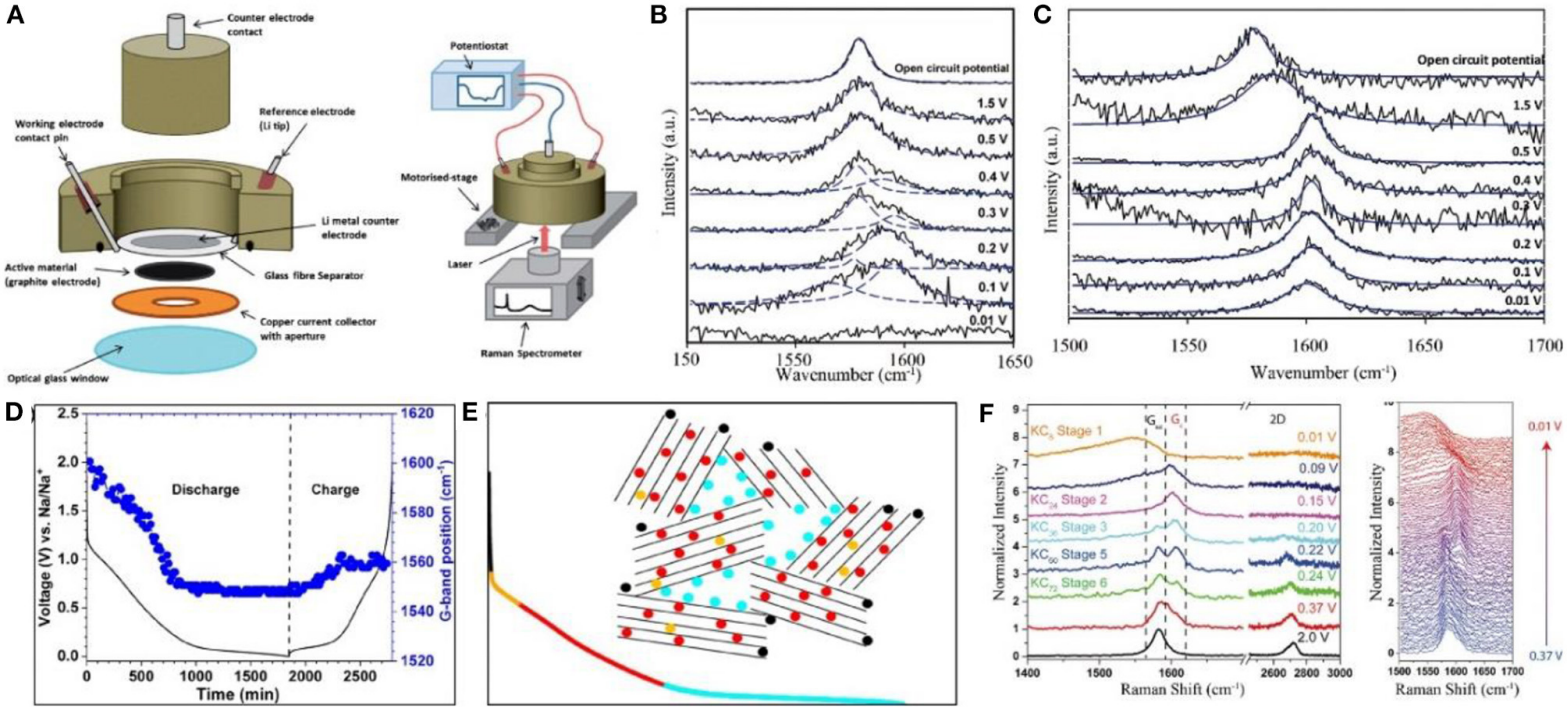
**(A)** A schematic of the Raman cell assembly and in operation (Sole et al., 2014). **(B,C)** *In-situ* Raman spectra of the few-layer graphene and single layer graphene electrodes during electrochemical lithiation, respectively (Pollak et al., 2010). **(D)** Evolution of G-band position as a function of discharge and charge (Anji Reddy et al., 2018). **(E)** The discharge curve. The color code qualitatively represents different processes during discharge: Adsorption on surface sites (black), filling of defects in graphitic layers (orange), filling of the layers (red) and adsorption on nanopores (light blue) (Anji Reddy et al., 2018). **(F)** Selective Raman spectra taken at different states of charge and the waterfall plot of all Raman spectra taken between 0.37 and 0.01 V (Share et al., 2016).

Optical spectroscopy is an effective technique to identify materials and determine their structures, chemical compositions, and relative contents. Since the energy level of atoms, ions, and molecules varies in different materials, the energy levels of absorbing/emitting photons are also characteristic. For carbon materials, the optical spectroscopy is mainly based on Raman spectra and FTIR. The former focuses on structural changes in carbon materials (Figure 3A), and the latter is applicable to characterize molecular functional groups, which can be used in conjunction with Raman spectra.

Generally, carbon materials demonstrate three main peaks at 1,336, 1,580, and 2,670 cm^−1^ in Raman spectra, corresponding names are D, G, and 2D bands, respectively. D band, generally associated with the disordered materials, is applied to characterize graphite edge planes and internal defects. G peak normally shows the structural information for the graphite layer, such as the changes of C=C bond, electron cloud density, etc. 2D band is usually related to the number of graphene layers, and its changes are more complicated.

D band usually appears at a potential lower than 1.0–0.6 V during cycling of graphite/Li cells, which generally occurs with the generation of the SEI film, and D band disappears when the SEI film becomes stable. It is difficult to observe the behavior of ion insertion into graphite through the changes of D band.

2D band existing below 0.15 V is mainly used to characterize the lithium intercalation mechanism. As ions intercalate into graphite, the graphite layer may bend and the electron cloud density between the graphite layers changes, resulting in a shift in 2D peak. Therefore, a reaction model for ions' insertion into graphite can be determined. For example, the 2D peak decreases from 2,681 to 2,611 cm^−1^ during the charging process, demonstrating that the graphite layer in the anode is bent after the ion insertion, corresponding with the Daumas–Hérold model (Sole et al., 2014).

The significant changes in the G band peak suggest the necessity of investigation on ion insertion mechanism (Figures 3B,C). In the absence of Li^+^ insertion, i.e., 1.5–0.5 V, the value of the G band is around 1,582 cm^−1^. With the beginning of lithium ion insertion into graphene layers, both the shoulder peak and the characteristic peak gradually raise up with the increase of the Li^+^ content (Figure 3C). Simlar with voltage, the peak position of the G band shifts with the amount of inserted ions. The G band signal for single-layer graphene starts moving at 1.5 V and becomes stable at 0.5 V (Figure 3B), reflecting that the G band in Raman spectra is sensitive to the reactions in carbon materials. Therefore, it can be concluded that the capacity of single-layer graphene is not contributed to by the ion intercalated mechanism but by the reaction between graphene and ions or the adsorption mechanism of graphene (Pollak et al., 2010).

Reddy et al. investigated the working mechanism of sodium-ion batteries with hard carbon as an anode material. There are a large number of defects in hard carbon, which shows a strong D band peak. During the initial discharge process, the D band peak becomes weak, proving the adsorption and reaction mechanisms of Na storage. Afterwards, the G band peak shifts to a low-frequency region and becomes stable at 0.2 V. This also proves that the adsorption mechanism is the final stage of the capacity contribution in sodium storage (Figures 3D,E) (Anji Reddy et al., 2018).

It was found that K^+^ were embedded in graphite without an initial dilution stage (Yoshino et al., 1987; Jian et al., 2015; Cohn et al., 2016) and no gradual peak shifting could be observed in Raman spectra. Pint et al. reported that the G band of graphene sheets moved to the high-frequency region of 1,589 cm^−1^ at 0.37 V for potassium ion batteries. At 0.15 V, only one peak at 1,589 cm^−1^ is identified. Subsequently, the peak began to evolve symmetrically and eventually generated a new peak. Therefore, three stages in K^+^ insertion into the graphene layers were proposed, namely KC_72_, KC_24_, KC_8_ (Share et al., 2016), and this mechanism is similar to Lithium intercalation into graphene sheets which was reported by Pollak et al. (2010) (Figure 3F).

By comparing graphene sheets with different layers, the failure mechanism can be predicted through the variation in tension of the single-layer material which is evaluated by the G band. Generally, failure of anode material is caused by structural changes, volume expansion, and damage to the SEI film, and Raman spectra has been proved to be an efficient technique for failure mechanism prediction (Zou et al., 2016).

FTIR, sensitive to polar groups, e.g., OH^−^, C=O, COO^−^, is a good technique to characterize changes of functional groups in carbon materials (Li et al., 2013). For example, *in-situ* FTIR can show the evolution of the groups in reduced graphene oxide (rGO) during the electrochemical process. The absorption strength of oxygen-containing groups is significantly weaker than that of pristine materials after the initial cycle, demonstrating that the capacity of rGO in the first cycle is mostly irreversible. The signal for the C=O group in the EC is clearly identified in the SEI film, proving that the EC molecule plays a great role in the SEI formation.

Fiber Evanescent Wave Spectroscopy, a cost-effective, real-time, non-destructive, and robust method to optically interrogate a harsh environment, is also designed as an *in-situ* characterization method for LIBs (Ghannoum et al., 2016). By quantitatively analyzing the reflectance of visible and infrared light (500–900 nm), a direct correlation between the state of charge and the measured reflectance is demonstrated for graphite anode in wavelengths ranging from 750 to 900 nm (near-infrared band). Based on this observation, a custom-designed Swagelok cell with etched optical fiber embedded between the graphite electrode and separator is assembled to measure the transmittance of graphite anode in the near-infrared band. This technique is expected to be developed as a unique inexpensive method to estimate the SOC of a LIB.

### *In-situ* Diffractive Techniques (Neutrons/X-Rays)

XRD and neutron diffraction (ND) are the most important techniques to study material structure. The orientation and intensity of the diffraction in the spatial distribution are closely related to the crystal structure. However, since the principle of XRD is that the X-ray interacts with electrons, the obtained scattering intensity in XRD patterns is proportional to the atomic number of elements, and a low sensitivity to light elements such as Li and C is delivered. Fortunately, scattering intensity of ND originated from the interaction between neutrons and nuclei is non-linearly related to nuclei, therefore ND is particularly suitable for probing the atomic arrangements of lithium and carbon during lithium intercalation. Hence, XRD is applied to provide information on the reversibility of the Li^+^ intercalation process through the structure changes of graphite while the ND is suitable for the determination of compositions of LiC_x_.

Ion-intercalated graphite would induce a series of phase transitions as well as the volume change. The former has guiding significance for developing new electrode materials while the latter provides valuable information for practical applications (capacity decay and safety issue). Graphite is composed of multiple layers of graphene, and the intercalation mechanism at the beginning of ion intercalation, during which ions would be uniformly intercalated into each layer or into specific layers, is a remaining debate. Fortunately, combining *in-situ* XRD and ND, the structural evolution and lithium intercalation behavior can be well-characterized.

**Figure 4:**
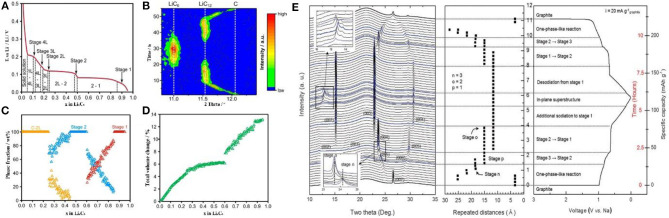
**(A)** The different lithium intercalation stages and phase transition regimes in the second cycle. **(B)** Evolution of the 002 reflection (equivalent to 002 for LiC_12_ and 001 for LiC_6_) during the second cycle. **(C)** Phase fraction, and **(D)** total volume change of graphite vs. lithium content (Schweidler et al., 2018). **(E)** In operando synchrotron X-ray diffraction analysis of the structural evolution of the ternary Na-ether-graphite system observed during electrochemical solvated-Na-ion intercalation and deintercalation into/out of graphite (Kim et al., 2015).

Dahn, Janek, and other groups demonstrated the phase diagram of Li_x_C_6_ and presented the single-phase and coexisting-phase region by *in-situ* XRD (Figures 4A–C) (Dahn, 1991; Schweidler et al., 2018). The phase region and the graphite volume expansion are interrelated: In stage 2 and stage 1, the volume of graphite remains constant and only the single-phase is observed. At the coexisting-phase region, graphite is in an indeterminate state and tends to be stable. In order to achieve stability under Li intercalation conditions, the graphene layers in graphite must be in the same chemical environment (Mathiesen et al., 2019). Therefore, there are only two possibilities: (1) lithium intercalates into each layer indiscriminately; (2) lithium intercalates into only one layer in every two layers, so that all graphenes are in the same chemical state (Figure 4C). In the dilute region, lithium inserts into the graphene interlayer, enlarging the spatial distance of the graphene layers, thus the volume expands in *c* axis. During the transition from stage 2 to stage 1, more space is needed for lithium storage and thus lithium intercalates into the unoccupied layers accompanied by further expansion in volume (Figure 4D). This mechanism can explain well why the battery is allowed to be discharged and charged to state of charge (SOC) 20 and 80%, respectively. The reasons are as follows: (1) It is necessary to retain certain lithium ions in graphite layer during discharge, ensuring the reversible lithium intercalation/deintercalation without destroying the graphite structure; (2) when charged to SOC of 100%, the risk of Li metal deposition on the surface of the negative electrode is greatly increased, resulting in safety issues; it is likely to cause the deposition of the Li metal in the negative electrode, which may cause serious safety issues; (3) when the battery is operated between SOC of 20–80%, the graphite experiences only a small volume expansion, which is beneficial to the stability of the battery.

For LIBs, NIBs, and PIBs with different electrolyte systems, *in-situ* XRD research is important to understand the battery operating mechanism. By collecting the signal of crystal phases, the structure changes of the electrode material during the charging/discharging process can be well-detected. For example, Kang et al. found that [Na–ether]^+^ complexes would be reversibly intercalated into carbon materials and the intercalation of solvated Na^+^ followed a stage process. After several intercalation/deimtercalation cycles, Na_x_*C*_y_ a single phase of Na_x_C_y_ is observed at Na/C ratio of 1/72 and the stage 1 compound is presented as a mixture with Na/C ratio from 1/28 to 1/21 (Figure 4E) (Kim et al., 2015).

Although some valuable phenomena have been observed, the structural evolution of Li^+^ embedded in graphite is still not fully understood due to the weak scattering of X-rays by Li and C, especially at a low lithium content. In this regard, neutron diffraction, which shows a strong diffraction intensity for light elements, can be employed as a powerful supplementary tool to track the details of the intercalation/deintercalation process.

**Figure 5:**
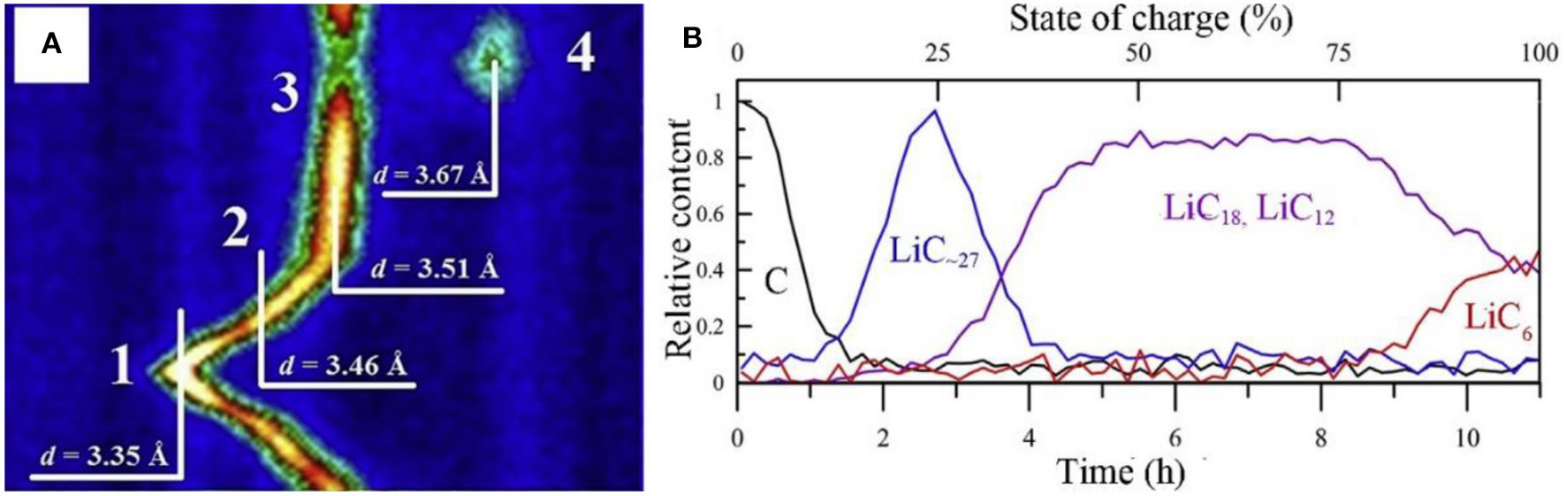
**(A)** 2D diffraction pattern region characterizing structural processes in LFP-battery anode. Initial state of the anode graphite without lithium (1, d = 3.35 Å), and sequentially emerging phases LiC_~27_ (2, d = 3.46 Å), LiC_18_, LiC_12_ (3, d = 3.51 Å), and LiC_6_ (4, d = 3.67 Å) for battery. **(B)** Content of different LiC_x_ phases of battery in course of one charge cycle as a function of time (lower scale) or SOC (upper scale) recovered from relative changes in intensity of corresponding diffraction peaks (Bobrikov et al., 2014).

In the initial process of intercalation, the LiC_x_ intermediate phase is hardly confirmed by XRD due to the low content. Based on the hexagonal structure LiC_x_, *c* lattice parameter of 10.42 Å is considered to be related to three layers of lithiated graphene (Pang et al., 2014). ND displays an identifiable scattering intensity even at a very small amount of LiC_x_. Nanda et al. revealed the existence of LiC_12_ phase in the final stage of lithium insertion in a commercial battery, proving that full intercalation of graphite is prohibited under real working condition. More specifically, Pang et al. investigated a LiNi_1/3_*Co*_1/3_Mn_1/3_*O*_2_/graphite full cell with 91 mol% LiC_6_ and 9 mol% LiC_12_ at an overcharge potential of 4.5 V (Pang et al., 2014). The detailed information of lithium intercalation/deintercalation and the transformation of LiC_x_ intermediate phase can be tracked through neutron diffraction. Bobrikov et al. studied the changes of graphite by ND. It is found that the initial increase in *d* space is resulted from the formation of LiC_~27_.Further Li^+^ intercalation leads to the formation of LiC_18_ phase. From the phase of LiC_18_ to LiC_12_, there is no obvious change in *d* space and it is considered to be due to the redistribution of lithium in the basal planes of graphite (Figure 5A). Based on the strong scattering data, one can illustrate the content of different LiC_x_ phases at different states of charge (Figure 5B) (Bobrikov et al., 2014).

Since XRD shows advantages in the determination of unit cell parameters while neutron diffraction is more sensitive to light elements, there are still certain differences between these two *in-situ* techniques in characterizing material structural changes. The structural changes of graphite anode during cycling were investigated through *in situ* neutron diffraction and *in situ* high energy synchrotron XRD by Sharma et al. (2010) and He et al. (2013), respectively. In both works, a two-phase region composed of the lithiated graphite phase and LiC_6_ was identified. According to phase changes shown by neutron diffraction, it is inferred that Li may be trapped in the SEI layer results in minimal structural changes to the lithiated graphite anode across the constant cell voltage regions of the electrochemical cycle. In the *in situ* XRD work, the relationship between the variation of d-spacing and the lithium content in graphite has been explored, and it is found that the dynamic mechanism of lithium intercalation into graphite may deviate from the traditional stage mechanism at a higher rate. It is noted that XRD shows more advantages than neutron diffraction for Na and K ions with larger atomic numbers.

Small-angle neutron scattering (SANS) is a technique to study the internal mesoscale structure of materials by elastic neutron scattering. Due to its high sensitivity to light elements, the lithium inercalation/deintercalation process can be visually observed during charging/discharging. During lithium intercalation, the relationship between lithium intercalation and voltage can be deduced due to the different neutron absorption intensity of lithium and graphite (Zhou et al., 2016).

### *In-situ* SAXS/SANS

**Figure 6:**
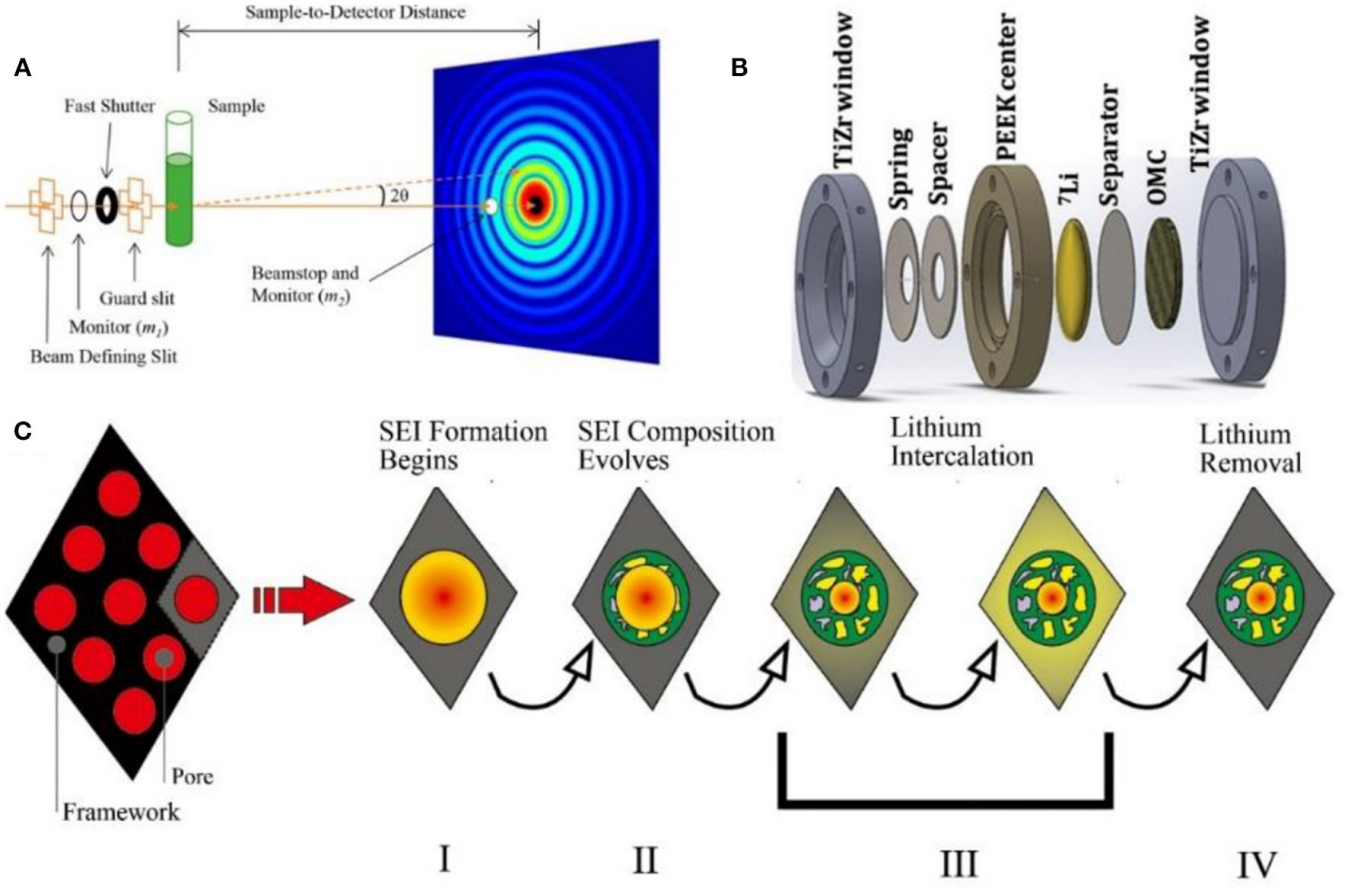
**(A)** Schematic illustration of the various stages of the initial charge/discharge cycle for the non-deuterated cell (Li et al., 2016). **(B)** Schematic view of the operando SANS cell (Jafta et al., 2019). **(C)** Schematic illustration of the various stages of the initial charge/discharge cycle for the non-deuterated cell, including: Stage I (pore filling and surface adsorption of solvated lithium during discharge), Stage II (SEI formation), Stage III (lithium intercalation), and Stage IV (lithium removal during charging) (Bridges et al., 2012).

When X-rays pass through a sample with nano-scale density inhomogeneities inside, the scattering phenomenon occurs within a small angle range (2θ ≤ 5°) close to the original beam, which is called small-angle neutron scattering (SAXS) (Figure 6A). Since the signal is a statistical result of many scatterers affected by particle size, shape, dispersion, orientation, and electron density distribution, SAXS is an important method to study the submicroscopic internal structure of materials.

SAXS is a superior technique to study the changes of pore structure. Synchronous light source with high energy X-rays enables SAXS to *in-situly* characterize the porous carbons in lithium-ion batteries, such as the entry of electrolytes, chemical reactions in the pores and the changes of pores. Csencsits et al. investigated the lithium storage during the charging/discharging process through continuous SAXS and no evident structure change was observed, suggesting that the electrolyte does not enter the porous material and would not react with the inside material (Sandí, 1999).

Generally, porous carbon materials exhibit high specific capacity during ions storage and there is no clear explanation for this high capacity. In Dhan's work, no changes in the pore structure of porous carbon materials has been observed during sodium intercalation. Instead, when the cell is discharged close to 0 V, sodium begins to deposit in the micropores to form metallic sodium, which blocks the micropores. During the charging process, the metallic sodium migrates away and the pores are gradually released. This work proves that the metal deposition in carbon materials is reversible (Stevens and Dahn, 2000b).

The basic principle of SANS is similar to that of SAXS (Figure 6B), while SANS is sensitive to the light elements, such as H, O, Li, etc. (Bridges et al., 2012; Zhou et al., 2016). This advantage is beneficial in determining the components and position of light elements in materials. In particular, *in-situ*, SANS is suitable for observing the interface between the carbon materials and the electrolyte, as well as the structural changes of the bulk material. Therefore, SANS is applied to study the SEI growth, components and lithiation gradients in carbon materials.

SANS can observe the framework changes of hard carbon in different electrolyte systems. During the formation of SEI film in the first cycle, the graphite interlayer will be enlarged due to the intercalation of solvated lithium ions into graphite, causing an increase of about 5%. After the SEI film is well-grown, the volume changes during cycling would be <1% (Bridges et al., 2017). It is noted that SEI film growth occurs in all pores in a hard carbon framework (Figure 6C). Besides, the results show that SEI is composed of various lithium containing salt, such as Li_2_CO_3_ and/or (CH_2_OCO_2_Li)_2_. As the charge/discharge cycle progresses, the content of the inorganic phase, such as Li_2_O, LiOH, LiF, etc. gradually increases. It is also observed that the intercalation of lithium occurred at a lower potential which was the dominant scattering process (Bridges et al., 2012). Jafta et al. found that the SEI formation followed a similar mechanism in both diluted and high concentration electrolytes (Jafta et al., 2019). However, due to the high viscosity of the high concentration electrolyte, the filling of the micropores occurs only at a low potential. Moreover, the formed SEI film is thinner in high concentration electrolyte since the SEI is formed by the decomposition of Li salt instead of the organic solvents.

Imaging, phase changes of pores, and crystals, as well as the component detection are important techniques to understand the working mechanism of batteries. They also provide important reference for improving the consistency of industrial batteries and battery failure analysis.

### *In-situ* TEM

**Figure 7:**
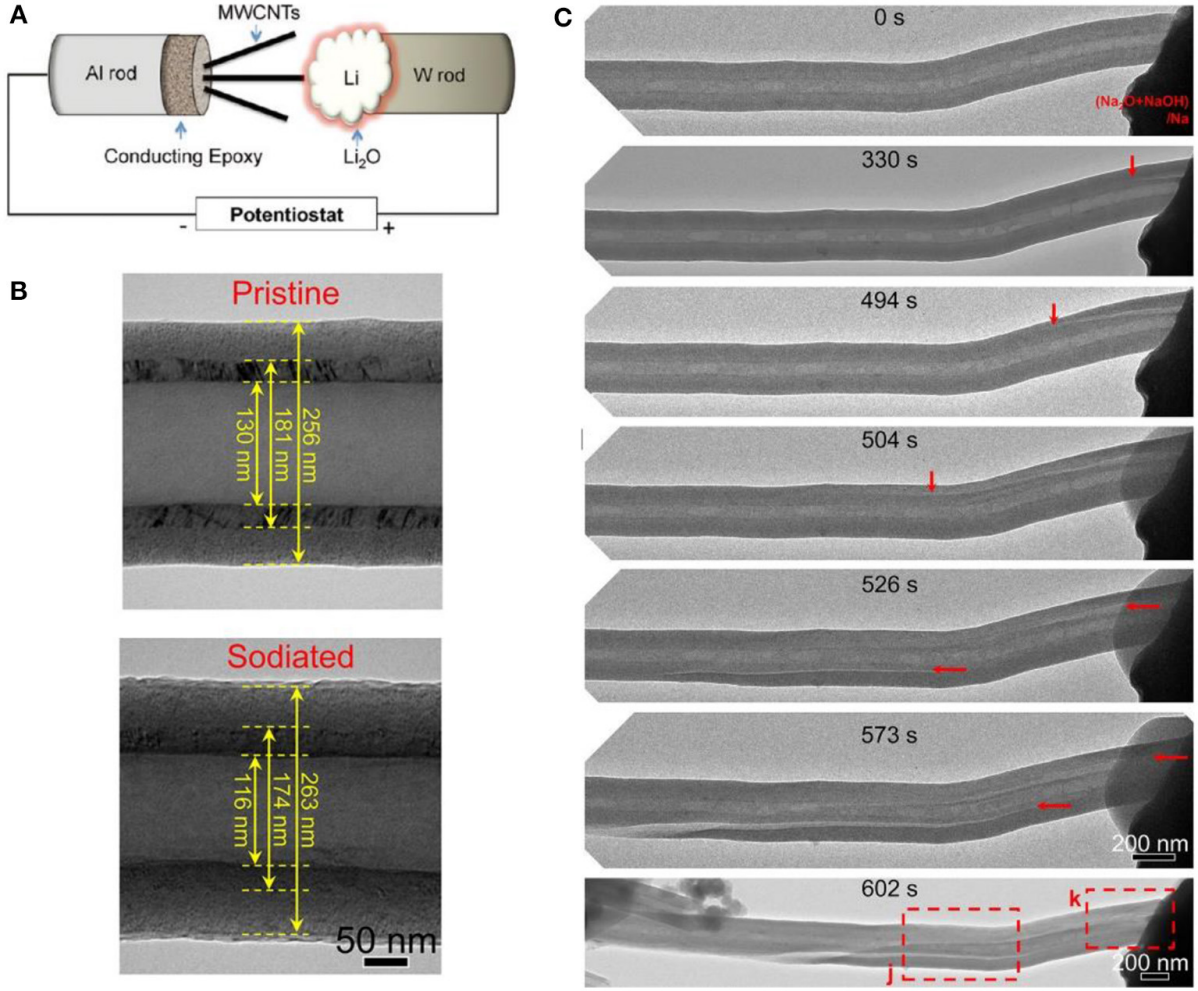
**(A)** Schematic illustration of the *in-situ* TEM experimental setup. **(B)** Structure changes of a bilayer CNF during sodiation. **(C)** Sodiation-induced crack nucleation and propagation in a hollow bilayer CNF (Liu et al., 2014).

TEM is the projection of an accelerated and concentrated electron beam onto a thin sample and the electron collides with atoms to change its direction, resulting in solid angle scattering. Since the scattering angle is related to the density and thickness of the sample, the images are formed with different brightness and darkness, and displayed on a imaging device after being enlarged (Figure 7A). The TEM images are very sensitive to the homogeneity and spatial change of the characterized samples. As all known, electrode materials are often accompanied by complex interface evolution, new phases formation, structures change, etc. during charge and discharge process. Therefore, *in-situ* TEM is emerging as a powerful tool in revealing the underlying reaction mechanisms between the intercalated ions and carbon host, observing the change of SEI film and microstructure during cycling (Wen et al., 2014).

One of the basic functions of *in-situ* TEM is to observe the change of layer spacing. After sodium intercalation, the graphite layer spacing increases from 0.38 to 0.40 nm (Wang et al., 2019). The size of the carbon nanotubes and few-layer graphene sheets increases from 3.4 to 3.6 Å, with a 5.9% diameter expansion (Liu et al., 2011). Due to its one-dimensional structure, it is much easier to characterize carbon fiber *in-situ* (Figure 7B).

TEM can also capture the information about the formation of new phases during the electrochemical process. During lithiation, a Li_2_O layer would be formed in several layers on the surface of graphene nanoribbons. However, this formed Li_2_O layer does not completely transform to freely migrating lithium ions during delithiation. As a result, part of Li_2_O deposits as the component of SEI film (Liu et al., 2012). Direct observation of irreversible oxides, such as Na_2_O, can be used to estimate the cause of battery capacity loss, especially in the materials with large specific surface area or abundant hydroxyl groups (Liu et al., 2011; Wan et al., 2016).

rGO has a large specific surface area, multiple defects, and a complex ionization mechanism. Hu et al. studied the electrochemical behavior on rGO through *in-situ* TEM. At voltage between 2 and 0.15 V, Na^+^ is mainly adsorbed on the surface of defects of rGO. Below 0.15 V, rGO begins to swell and sodium ions are inserted into the graphite layer (Wang et al., 2019). Besides, it is also occasionally observed that metallic sodium clusters with the sizes of 10 nm are deposited on the surface and defects of rGO, leading to a high reversible capacity. In half-cells, rGO shows a reversible specific capacity of up to 450 mAh g^−1^. Due to the low volume expansion, rGO can cycle 750 cycles at high rate (Wan et al., 2016).

Generally, failure of carbon material is accompanied by instability of the material structure. After lithium intercalation, MWCNTs become brittle and the outer carbon structure begins to collapse when stretched (Liu et al., 2011). This collapse would lead to the growth of flaws in the SEI film. Subsequently, the collapse begins to spread along the axis. To provide mechanistic insights into the electrochemical reaction, microstructure evolution and mechanical degradation of carbon-based anodes during sodiation and potassiation, individual carbon nanofibers (CNFs) was studied by *in situ* TEM (Figure 7C) and this revealed that the mechanical degradation of CNFs takes place through the formation of longitudinal cracks near the c-C/d-C interface during sodiation and potassiation (Liu et al., 2014).

In addition to the volume change and material analysis, *in-situ* TEM also provides information on the rate and uniformity of ion transport in carbon materials (Shan et al., 2014).

### *In-situ* NMR

**Figure 8:**
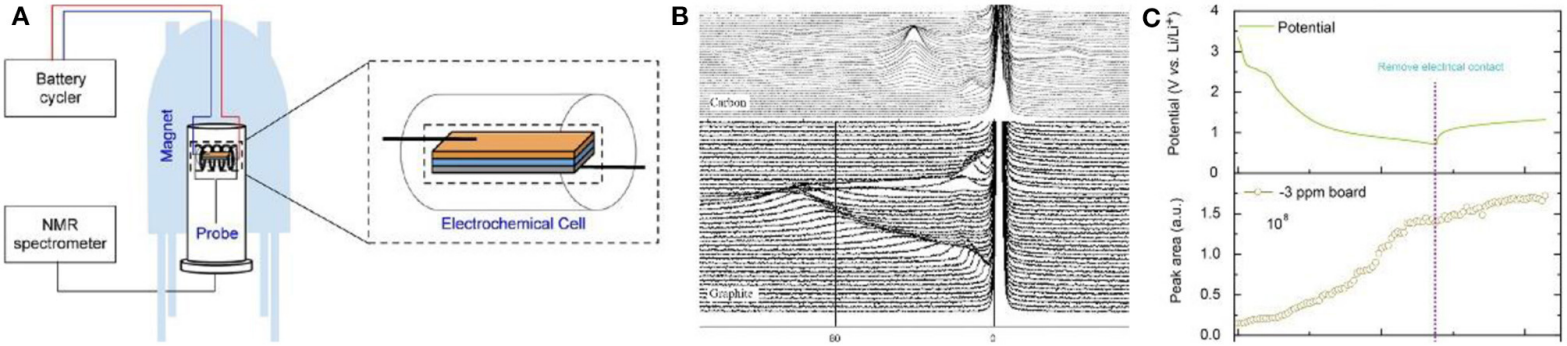
**(A)** Schematic illustration of the *in-situ* NMR experimental setup. **(B)** *In-situ* ^7^Li NMR during the first galvanostatic cycle: top picture, lithium in graphite; bottom picture, lithium in the carbon/carbon composite (Letellier et al., 2006). **(C)** Potential–time plot to demonstrate the potential evolution after remove the electrical contact from rGO cell (top); *In-situ* NMR peak area evolution of the −3 ppm board (bottom) (Tang et al., 2016).

NMR, which provides information about the number and type of chemical groups in the molecule, is an important technique to determine organic and inorganic compounds in battery at different states of charge (Figure 8A). In a single NMR spectrum, different lithium compounds, such as lithium salt in the electrolyte, lithium compounds in the SEI film and lithium intercalated into graphite, can be identified separately. And thus, the state of lithium during the charge/discharge process can be clearly observed by *in-situ* NMR (Gerald et al., 2001).

For NMR testing, both ^6^Li and ^7^Li can be used, however, ^7^Li is more sensitive to discriminate in the spectrum. In a spectrum, lithium in SEI films and electrolyte exists in the 0 ppm while metallic Li is located at near 263 ppm. During the intercalation process of lithium into carbon materials, the peak for LiC_x_ gradually shifts from 0 ppm to the position for Li metal (Letellier et al., 2003). Letellier et al. did not observe independent phases in *in-situ* ^7^Li NMR, indicating that the intercalation of lithium into graphite is relatively continuous, while LiC_x_ species at each stage can be distinguished (Figure 8B) (Stevens and Dahn, 2000b; Bridges et al., 2017).

SEI film plays an important role in carbon anode materials and its growth mechanism and composition are crucial to battery design. Loh et al. studied the lithium compounds in SEI and found that their content gradually increased during the first discharge process and then was basically stable in the following charge process (Figure 8C). It is worth noting that the changes during high self-discharge was also intuitively observed by *in-situ* NMR (Tang et al., 2016). Unfortunately, only the specific composition and formation of the SEI film on the carbon surface were investigated by NMR, the detailed mechanism was not discussed. Later, the surface of Si anode was analyzed through NMR C spectrum by Grey et al., confirming that NMR can be applied to speculate the specific decomposition mechanism of the electrolyte on the carbon surface as well as the influence of the electrolyte additive on the SEI film formation (Jin et al., 2017).

Different from a single technique, multiple techniques can provide more information to better reflect the internal structural changes. To promote better fundamental understanding of K^+^ storage behavior in graphite, *in situ* Raman mapping and *in situ* X-ray diffraction (XRD) characterizations, in combination with density functional theory simulations are carried out to correlate the real-time electrochemical K^+^ intercalation/deintercalation process with structure/component evolution. The experimental results, together with theoretical calculations, reveal that the reversible K ion intercalation into graphite follows a staging transition (Liu J. et al., 2019).

### Summary of *in-situ* Techniques for Carbon Materials

**Figure 9:**
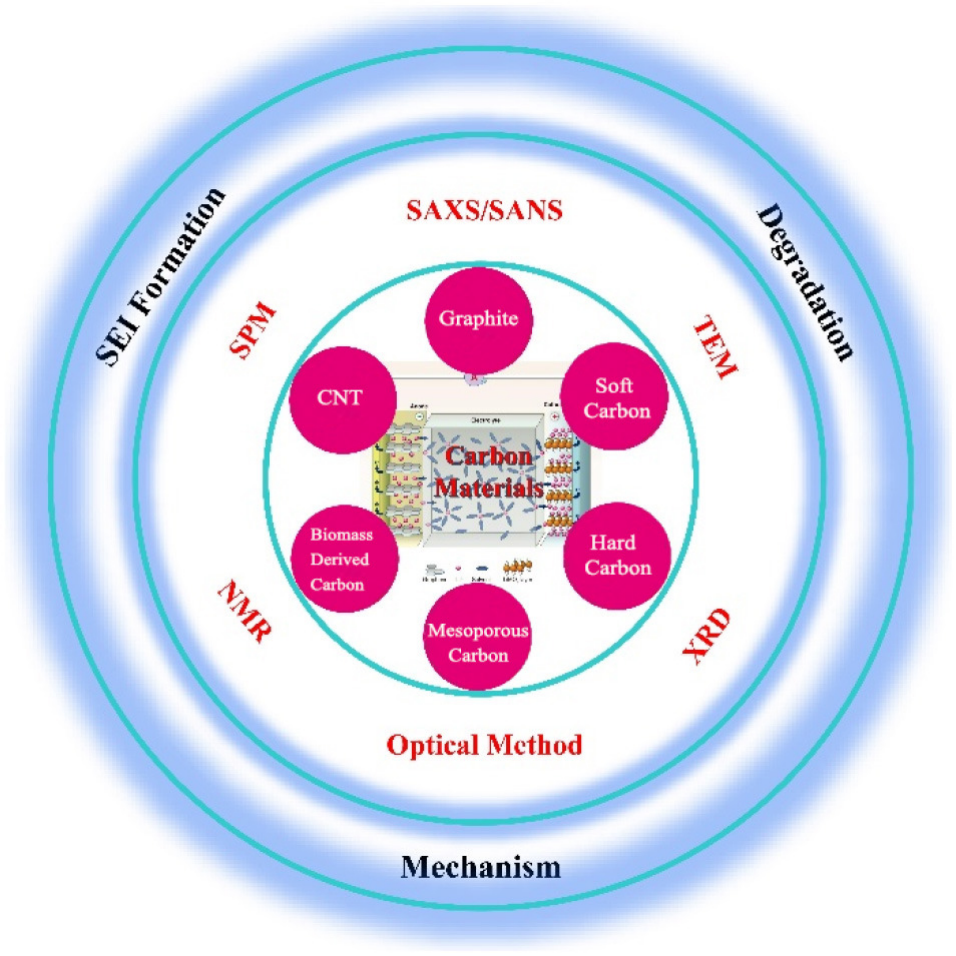
Scheme of *in-situ* techniques for the studies of carbon anode in alkali metal ion batteries (Dunn et al., 2011).

So far, carbon-based materials are the most ideal and widely used anode materials for alkali ion batteries. With the increasing requirements on the performance of commercial batteries, such as energy density, operating temperature range and rapid charging/discharging, developing new carbon-based anode materials is one of the most concerned issues. Figuring out the ion diffusion behavior of the battery under real working conditions, and understanding the working mechanism of the active materials and the changes of the interface are of great significance for the designing new materials and improving battery performance. However, the mechanism of different carbon materials, the structural changes of the same material at different working conditions, the formation/transformation of chemical compounds are extremely complicated, and it is difficult to characterize them with conventional methods (Figure 9).

With *in-situ* technologies, changes of components and material structure in the battery can be characterized operandoly while the electrochemical reaction is proceeding. However, it is difficult to deduce the working mechanism based on the data obtained by a single characterization technique. In terms of the mechanism characterization of alkali metal ion batteries, SPM and TEM are suitable for observing the growth of SEI film on the surface of carbon materials, Raman spectroscopy can observe the changes of carbon materials during the formation of SEI film, FTIR, and NMR are used to determine the chemical composition of the SEI film, XRD and ND are applied to study intercalation/deintercalation behaviors of alkali ions into/from carbon materials, SAXS and SANS mainly observe the structural changes of microporous or mesoporous materials which are of great benefit to help understand the ions intercalation and the SEI formation, and XRD, TEM, and SANS are often used in the study of material failure. By combining the observation of these characterization techniques, a true working mechanism of alkali metal ion batteries would be better understood and deduced.

## Summary and Perspectives

In conclusion, the recent research on the understanding the working behavior of carbon materials in alkali ion batteries through *in-situ* techniques has been summarized. The ion intercalation mechanism, SEI formation and degradation of carbon materials in anode have been explored using *in-situ* techniques including SPM, optical spectroscopy, XRD, SAXS/SANS, TEM, and NMR. Nonetheless, except for graphite, other types of materials are just partially studied. Hence, more details of the different carbon materials need to be investigated in order to provide guidance for future material development. It is noted that there are many studies on graphite anodes and their mechanism is relatively clear. However, the mechanism for new carbon-based materials is still controversial. Research on these new carbon materials can provide guidance for the development of new carbon-based anode materials.

As the anode material for the ion batteries, the specific capacity of graphite is limited by the ion intercalation mechanism. To increase the specific capacity of carbon-based materials, it is necessary to introduce a different capacity contribution mechanism. However, the new mechanisms, such as defect adsorption and ions deposition, lead to lower initial Colombic efficiency and thus difficulty in practical application in commercial batteries. Therefore, improving the initial Colombic efficiency and stability of carbon materials is the basis for designing new materials. As is well-known, the adsorption of ions in the defect sites and the deposition in the pores can greatly increase the specific capacity of the carbon based anode materials. However, there are still a large number of phenomena that need to be determined clearly, such as the influence of different kinds of defects, contribution of pore size and distribution to capacity, as well as the exact reaction process. Addressing these issues is essential for the design of practically applicable high specific capacity anode materials.

Analysis and understanding of chemical reactions, degradation mechanisms and thermal failure mechanisms ion batteries play particularly important roles for the development of confidence electrode materials. The material structural transformation during the charge/discharge process, the redox process, the transformation of SEI formation, and other side reactions that occur in the battery can be monitored by *in-situ* techniques. However, most of these *in-situ* techniques suffer from poor penetration and small characterization depth, as a result, *in situ* devices with complex structures need to be specially designed. Therefore, the development of non-destructive *in situ* techniques to directly monitor the structural changes in commercial batteries is still urgently needed. In addition, a single *in situ* characterization only evaluates specific signals which makes it difficult to fully reflect the behaviors inside the battery. To fully understand the behavior of materials under working conditions, multiple *in situ* techniques are required. Therefore, evaluation of the internal behaviors via multi-techniques under the same working conditions is the focus of future.

*In situ* techniques exhibit unparalleled advantages in mechanism research and characterization of reaction behavior. With the application of more *in situ* techniques and the maturity of technology, more details about the real working behaviors in alkali metal ion batteries will be observed. And these new findings will enhance the understanding of alkali metal ion batteries and promote the development of new electrode materials.

## Author Contributions

RD and YalH collected the articles and wrote the first manuscript. GL and QL organized references and revised the manuscript. TW organized the figures. YanH and YL modified the format and revised the manuscript. HH revised the manuscript, approved the final version, and supervised the whole work. All authors contributed to revise the manuscript, approved the final version, and agreed to be accountable for all aspects of this work.

## Conflict of Interest

The authors declare that the research was conducted in the absence of any commercial or financial relationships that could be construed as a potential conflict of interest.
